# Prognostic and Predictive Significance of *MYC* and *KRAS* Alterations in Breast Cancer from Women Treated with Neoadjuvant Chemotherapy

**DOI:** 10.1371/journal.pone.0060576

**Published:** 2013-03-26

**Authors:** Cynthia Brito Lins Pereira, Mariana Ferreira Leal, Carolina Rosal Teixeira de Souza, Raquel Carvalho Montenegro, Juan Antonio Rey, Antônio Alberto Carvalho, Paulo Pimentel Assumpção, André Salim Khayat, Giovanny Rebouças Pinto, Sâmia Demachki, Marília de Arruda Cardoso Smith, Rommel Rodríguez Burbano

**Affiliations:** 1 Mastology Unit, Ophir Loyola Hospital, Belém, PA, Brazil; 2 Department of Orthopaedics and Traumatology, Federal University of São Paulo, São Paulo, SP, Brazil; 3 Human Cytogenetics Laboratory, Institute of Biological Sciences, Federal University of Pará, Belém, PA, Brazil; 4 Research Unit-Unidad de Investigación, Hospital Universitario La Paz, Madrid, Spain; 5 Nucleu of Research in Oncology, João de Barros Barreto University Hospital, Federal University of Pará, Belém, PA, Brazil; 6 Department of Biomedicine, Federal University of Piauí, Parnaíba, PI, Brazil; 7 Genetics Division, Department of Morphology and Genetics, Federal University of São Paulo, São Paulo, SP, Brazil; Sanjay Gandhi Medical Institute, India

## Abstract

Breast cancer is a complex disease, with heterogeneous clinical evolution. Several analyses have been performed to identify the risk factors for breast cancer progression and the patients who respond best to a specific treatment. We aimed to evaluate whether the hormone receptor expression, *HER2* and *MYC* genes and their protein status, and *KRAS* codon 12 mutations may be prognostic or predictive biomarkers of breast cancer. Protein, gene and mutation status were concomitantly evaluated in 116 breast tumors from women who underwent neoadjuvant chemotherapy with doxorubicin plus cyclophosphamide. We observed that MYC expression was associated with luminal B and HER2 overexpression phenotypes compared to luminal A (p<0.05). The presence of *MYC* duplication or polysomy 8, as well as *KRAS* mutation, were also associated with the HER2 overexpression subtype (p<0.05). MYC expression and *MYC* gain were more frequently observed in early-onset compared to late-onset tumors (p<0.05). *KRAS* mutation was a risk factor of grade 3 tumors (p<0.05). A multivariate logistic regression demonstrated that *MYC* amplification defined as *MYC*/nucleus ratio of ≥2.5 was a protective factor for chemotherapy resistance. On the other hand, age and grade 2 tumors were a risk factor. Additionally, luminal B, HER2 overexpression, and triple-negative tumors presented increased odds of being resistant to chemotherapy relative to luminal A tumors. Thus, breast tumors with *KRAS* codon 12 mutations seem to present a worse prognosis. Additionally, *MYC* amplification may help in the identification of tumors that are sensitive to doxorubicin plus cyclophosphamide treatment. If confirmed in a large set of samples, these markers may be useful for clinical stratification and prognosis.

## Introduction

Several analyses have been performed to identify the risk factors for breast cancer (BC) progression. The histological response to preoperative chemotherapy is one of the most reliable predictors for prognosis of BC patients. Many different chemotherapy regimens have been applied in the preoperative setting. However, the identification of patients who respond best to a specific treatment is still critical to the appropriate management.

Some markers have been described as useful factors for prognostic evaluation or predicting therapeutic response. Several studies demonstrated that lack of estrogen (ER) and/or progesterone (PR) receptors predicts for chemosensitivity [1]. On the other hand, it has been proposed that HER2 (c-erbB2) is a predictor factor for either resistance or sensitivity to different types of chemotherapeutic agents. However, the literature results are still controversial, especially concerning the response to anthracyclines [2].

ER, PR and HER2 had been used to classify tumors according to luminal A (ER+/HER2−), luminal B (ER+/HER2+), HER2 overexpression (ER−/HER2+), and triple-negative (ER−/PR−/HER2−) molecular subtypes. HER2 overexpression and triple-negative are more aggressive and present poor prognosis than the luminal subtypes [3], [4]. Moreover, molecular phenotypes have become increasingly valuable in guiding treatment decisions. However, these markers remain imperfect tools and, therefore, new prognostic and predictive factors are still required to optimize treatments among BC patients [5].

MYC acts as a downstream target of HER2-driven proliferative signals in BC cells *in vitro* [6] and may be regulated by ER or PR contributing to different cell phenotypes [7]. MYC plays a role in the regulation of cell growth and proliferation, metabolism, differentiation, apoptosis, and angiogenesis [8]. *MYC* amplification and its protein overexpression have been found in about 15% and 40% of BC, respectively [9]. Due to the elevated frequency of alteration, it has been advocated that MYC is involved in BC development and progression [10].

Activation of HER2 induces activation of RAS, which enhances the accumulation of MYC activity by stabilizing the MYC protein [1], [11]. In a transgenic mouse model, the synergistic effects of *Myc* and the mutant *Kras* leads to breast tumor formation, maintenance, and recurrence [12]. These data suggest that the *KRAS* mutation may have a role in breast carcinogenesis.

In the present study, we evaluated the hormone receptor (HR) expression, *HER2* and *MYC* genes and their protein status, and *KRAS* codon 12 mutations in BC from women who underwent neoadjuvant chemotherapy, as well as their associations with clinicopathological features and chemotherapy response.

## Methods

### Patients and tumor samples

During the period from 2005 to 2011, 116 females were selected from a cohort of patients with locally advanced invasive ductal carcinoma who underwent therapeutic surgery for a first incidence of BC. All tumors were at stage III according to TNM staging [13]. Cardiac problems, presence of distant metastasis, pregnancy or lactation were exclusion criteria. The mean age of patients was 52±12 years (range of 31–83).

All patients were treated at Ophir Loyola Hospital (Pará, Brazil) and received Adriamycin (doxorubicin; 60 mg/m2) plus Cytoxan (cyclophosphamide; 600 mg/m2) by intravenous every 21 days for four cycles. The response to chemotherapy was based on the change in the primary tumor size on pre- and post-therapy. The tumor size was assessed by clinical palpation using a caliper. Tumors were classified as sensitive to chemotherapy if complete (macroscopic disappearance) or partial (at least a 50% reduction) response was achieved. Tumors were defined as treatment-resistant if no response (less than 50% reduction or less than 25% increase) or progression (at least a 25% increase or presence of new lesions) was observed.

Tumors were obtained by incisional biopsy before neoadjuvant chemotherapy. The tumor invasion and the nodal status were determined according to TNM staging [13]. The histological grade was assessed using the modified Scarff-Bloom-Richardson system [14].

Tumors were classified as luminal A, luminal B, HER2 overexpression, and triple-negative subtypes based on the ER, PR, and HER2 status [15]. We also classified data into tumors of early-onset (patients with ≤40 years of age) and late-onset (>40 years) [16], [17].

Tumors samples were formalin-fixed paraffin-embedded (FFPE). Sections of FFPE tissue were stained with hematoxylin-eosin for histological evaluation or used for immunohistochemical, FISH and PCR analyses.

The study was approved by the ethics committee of the Federal University of Pará, Brazil. Written informed consent with approval of the ethics committee was obtained from all patients prior to specimen collection.

### Immunohistochemistry

Immunohistochemistry was performed with primary monoclonal antibodies against ER (SAB4500810, Sigma, USA), PR (HPA004751, Sigma, USA), HER2 (Clone CB11, Life Technologies, USA) or MYC (clone 289–19510, Life Technologies, USA). Universal peroxidase-conjugated secondary antibody kit (DakoCytomation, USA) was used for the detection system and 3,30-diamino-benzidine/H_2_O_2_ (Dakocytomation, Denmark) was used as the chromogen. Positive ER, PR or MYC expression was defined as clear nuclear immunostaining in more than 10% of tumor cells [18], [19], [20], [21]. HER2 protein staining was scored as 0 (negative), 1+(weakly positive), 2+ (moderately positive) and 3+ (strongly positive) [2]. Double-blind analysis was performed on all samples.

A breast tissue sample from a male with gynecomastia was used as negative control. In addition, negative controls with primary antibody replaced with Tris-buffered saline were run with the patient slides.

### Dual-color FISH

*HER2* and *MYC* amplification was evaluated by dual-color FISH assay using Dako *ERBB2* FISH PharmDX™ Kit and *MYC*/CEN-8 FISH Probe Mix (Dako A/S, Denmark), respectively. FISH scoring was performed by counting fluorescence signals in at least 60 tumor cells. Double-blind analysis was performed on all samples.

For the detection of *HER2* amplification, the ratio of *HER2* signals to chromosome 17 (CEP17) signals was calculated according to the established guidelines. Patients were stratified depending on their *HER*2 gene status as: amplified if *HER2*/CEP17 ratio >2.2; not amplified if *HER2*/CEP17<1.8; equivocal if 1.8<*HER2*/CEP17<2.2 [2].

Since no established guideline was published, *MYC* amplification was defined using different cutoffs as per previously-criteria: 1) the ratio of *MYC* signals to chromosome 8 (CEP8) signals >2.2 [22], [23] as applied for the detection of *HER2* amplification; 2) *MYC*/CEP8 ratio≥1.3 (at least gene duplication) or *MYC*/CEP8 ratio<1.3 with polysomy 8 (3 or more copied of CEP8) [22], [23]; 3)>5 *MYC* copies/nucleus (high *MYC* gain) [23]; 4)≥2,5 *MYC* copies/nucleus, which included low *MYC* gain [23].

### Mutation analysis

**Figure 1:**
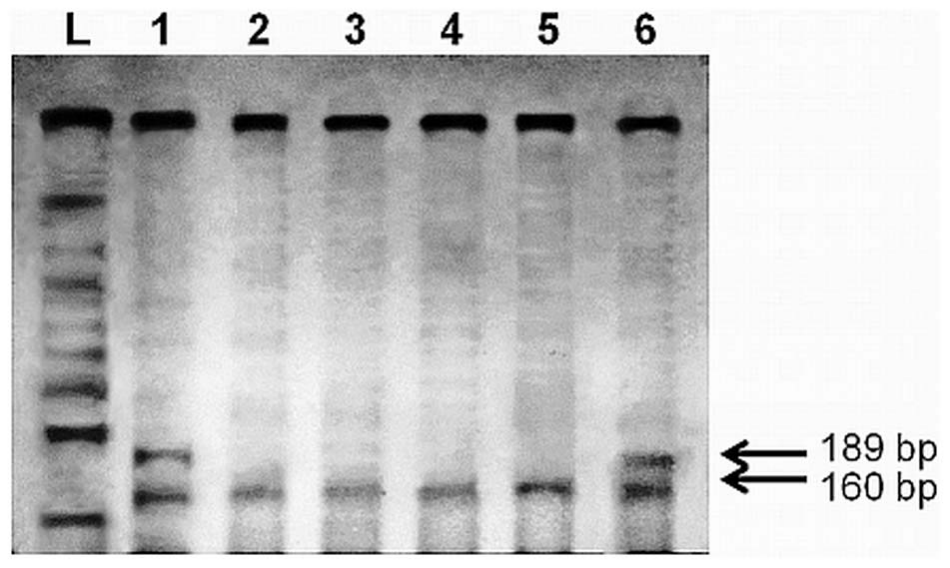
Mutation analysis by PCR-RFLP of *KRAS* codon 12. 1 and 6: tumors with mutation. 2–5: tumors without mutation. L: size marker.

DNA was purified using MagMAX^™^ FFPE DNA Isolation Kit (Life Technologies, USA). *KRAS* codon 12 point mutation was evaluated by PCR-RFLP as previously described [24]. PCR products were digested with endonuclease *BstO*I. The digestion products were electrophoresed on polyacrylamide gels with SYBR® Safe DNA Gel Stain (Life Technologies, USA) and visualized using blue light. The mutant-type (non-restricted PCR products) were 189 bp, whereas the wild-type products were 160 bp (Figure 1). The PCR products of muted *KRAS* were sequenced for confirmation of mutation using an ABI Prism® 377 DNA Sequencer (Life Technologies, USA).

A wild-type sample of peripheral blood lymphocytes from normal healthy individual and a colorectal cancer sample with codon 12 mutations were used as negative and positive controls, respectively. The controls were included in all experiments. All reactions were performed in duplicate.

### Statistical analyses

Cohen's kappa test (κ) was used to evaluate the concordance between the HER2 status by IHC and FISH. This rate was calculated considering negative cases (scores 0 and 1+ and no amplification), and positive cases (score 3+ and amplification). Patients with equivocal IHC or FISH results were not considered for this purpose [25]. Concordance was assessed by Fleiss' equally arbitrary guidelines, which characterize κ values over 0.75 as excellent, 0.40 to 0.75 as fair to good, and below 0.40 as poor [25].

In the remaining analyses, samples with *HER2*/CEP17>2.2 were classified as presenting *HER2* amplification and with scores of 2+ and 3+ as positive HER2 expression.

Logistic regression was used to evaluate the relationship between protein immunoreactivity, gene amplification or mutation, and clinicopathological features. IHC, FISH or PCR-RFLP results, as well as molecular phenotype, were considered dependent variables. Age was not added as a co-variable, since age did not differ between groups (by Student's T-test; data not shown).

A multivariate logistic regression in a forward stepwise approach (condition method) was used to identify variables that may help to predict chemotherapy resistance and to identify risk factors for grade 3 tumors. Age was also added as a dependent variable in the multivariate analyses.

In all analyses, p values less than 0.05 were considered significant. Odds ratio (OR) with 95% confidence intervals are shown.

## Results

### The protein and gene status and their relationships

For HER2, the percentage of concordant results between IHC and FISH was equal to 93.9%, with a statistically significant κ value of 0.833. The 5 discordant cases were classified as score 1+ by IHC and showed gene amplification by FISH. In addition, 6/32 of tumors classified as score 2+ presented *HER2*/CEP17 ration ≥2.2 (Table 1).

**Table 1:** HER2 protein and its gene status in the breast tumors.

IHC	FISH			Total
	Not amplified	Equivocal	Amplified	
1+	60 (89.55%)	2 (2.99%)	5 (7.46%)	67 (57.76%)
2+	23 (71.87%)	3 (9.38%)	6 (18.75%)	32 (27.59%)
3+	0 (0%)	0 (0%)	17 (100%)	17 (14.65%)
Total	83 (71.55%)	5 (4.31%)	28 (24.14%)	116 (100%)

IHC: immunohistochemistry; FISH: fluorescence in situ hybridization.

**Figure 2:**
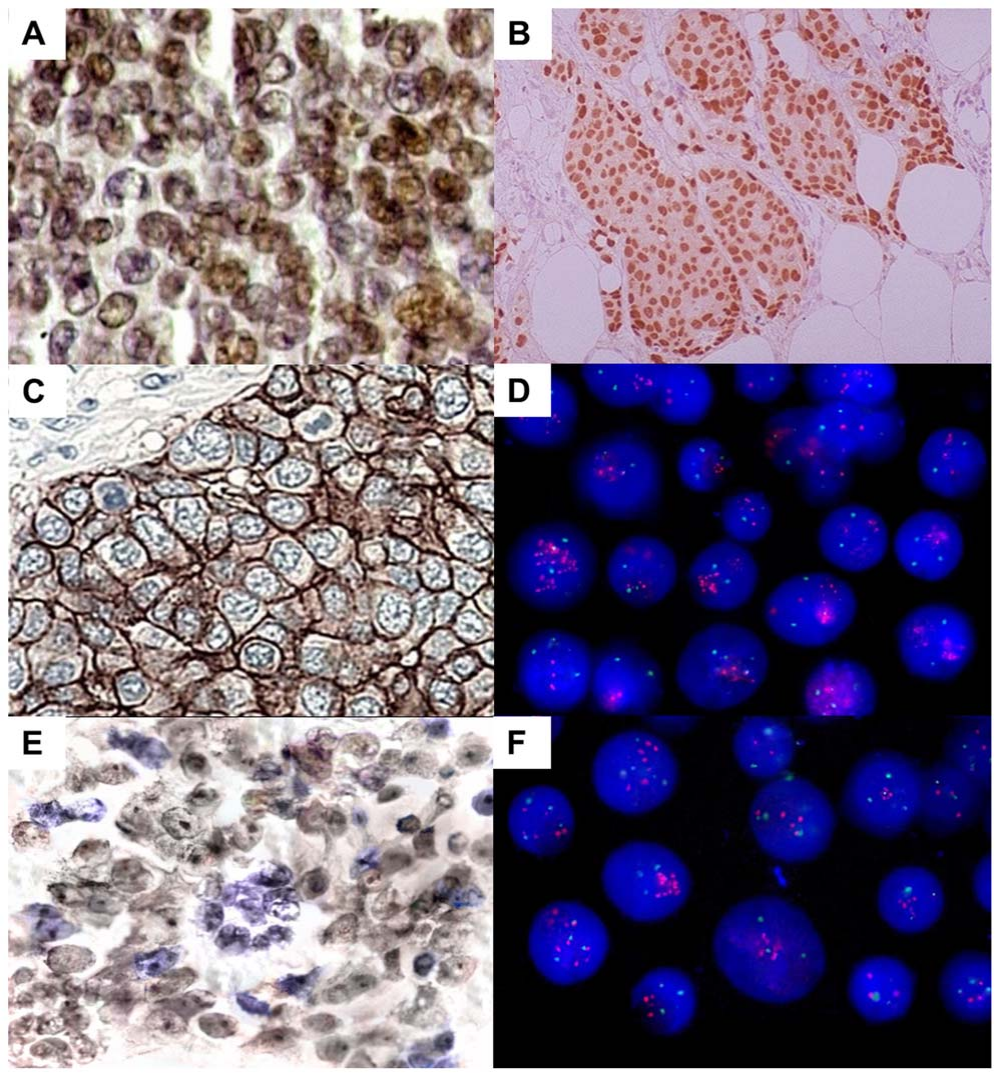
IHC and FISH analysis in breast tumors. a) Progesterone immunoreactivity (400x); b) Estrogen immunoreactivity (100x); c) HER2 immunoreactivity, score 3+ (400x); d) Interphase nuclei presenting two or more signals for chromosome 17 centromere (green) and *HER2* (red) (1000x); e) MYC immunoreactivity (400x); f) Interphase nuclei presenting two or more chromosome 8 centromere (green) and *MYC* signals (red) (1000x).

Table 2 presents the IHC results and Table 3 the FISH results. Figure 2 represents protein immunoreactivity by IHC and gene amplification by FISH assay. No ER-positive case was found without concomitant PR immunoreactivity. Therefore, tumors with ER and PR immunoreactivity were renamed as HR-positive cases for further analyses.

**Table 2:** Clinicopathological features by protein expression status.

Factor (N)	HR expression [N(%)]		OR (95% CI)	HER2 expression [N(%)]		OR (95% CI)	MYC expression [N(%)]		OR (95% CI)
	Negative	Positive*		Negative	Positive*		Negative	Positive*	
Age									
≤40 (27)	15 (12.93)	12 (10.34)	1.117 (0.470–2.655)	12 (10.34)	15 (12.93)	0.495 (0.207–1.182)	12 (10.34)	15 (12.93)	0.247 (0.100–0.610)**
>40 (89)*	47 (40.52)	42 (36.21)		55 (47.41)	34 (29.31)		68 (58.62)	21 (18.10)	
Grade									
1/2 (96)	46 (39.66)	50 (43.10)	0.230 (0.072–0.738)**	61 (52.59)	35 (30.17)	4.067 (1.433–11.537)**	67 (57.76)	29 (25.00)	1.244 (0.450–3.439)
3 (20)*	16 (13.79)	4 (3.45)		6 (5.17)	14 (12.07)		13 (11.21)	7 (6.03)	
Tumor invasion									
T1/T2 (9)	4 (3.45)	5 (4.31)	0.676 (0.172–2.656)	6 (5.17)	3 (2.59)	1.508 (0.358–6.352)	7 (6.03)	2 (1.72)	1.630 (0.322–8.264)
T3/T4 (107)*	58 (50)	49 (42.24)		61 (52.59)	46 (39.66)		73 (62.93)	34 (29.31)	
Lymph node metastasis									
Absent (6)	6 (5.17)	0 (0)	<0.001 (0)	2 (1.72)	4 (3.45)	0.346 (0.061–1.971)	3 (2.59)	3 (2.59)	0.429 (0.082–2.235)
Present (110)*	56 (48.28)	54 (46.55)		65 (56.03)	45 (38.79)		77 (66.38)	33 (28.45)	
Response to therapy									
Sensitive (50)	8 (6.90)	42 (36.21)	0.042 (0.016–0.113)**	42 (36.21)	8 (6.9)	8.610 (3.483–21.283)**	41 (35.34)	9 (7.76)	3.154 (1.318–7.547)**
Resistant (66)*	54 (46.55)	12 (10.34)		25 (21.55)	41 (35.34)		39 (33.62)	27 (23.28)	
HR expression									
Negative (62)	–	–	–	24 (20.69)	38 (32.76)	0.162 (0.070–0.373)**	37 (31.90)	25 (21.55)	0.379 (0.164–0.872)**
Positive (54)*	–	–		43 (37.07)	11 (9.48)		43 (37.07)	11 (9.48)	
HER2 expression									
Negative (67)	–	–	–	–	–	–	56 (48.28)	11 (9.48)	5.303 (2.255–12.473)**
Positive (49)*	–	–		–	–		24 (20.69)	25 (21.55)	

* Reference group for logistic regression analysis; ** Differentially expressed between groups, p<0.05. N: number of samples; OR: odds ratio; CI: confidence interval. HR: hormone receptor.

**Table 3:** Clinicopathological features and protein expression by *HER2* and *MYC* amplification status.

Factor (N)	*HER2* amplification [N(%)]		OR (95% CI)		*MYC* amplification [N(%)]		OR (95% CI)	*MYC* duplication or pol8 [N(%)]		OR (95% CI)	High *MYC* gain [N(%)]		OR (95% CI)	*MYC* gain [N(%)]		OR (95% CI)
	Negative (*HER2*/CEP17≤2.2)	Positive (*HER2*/CEP17>2.2)*			Negative (*MYC*/CEP8≤2.2)	Positive (*MYC*/CEP8>2.2)*		Negative (*MYC*/CEP8<1.3)	Positive (*MYC*/CEP8≥1.3 or pol8)		Negative (*MYC*/nucleus<5)	Positive (*MYC*/nucleus≥5)*		Negative (*MYC*/nucleus<2.5)	Positive (*MYC*/nucleus≥2.5)*	
Age																
≤40 (27)	17 (14.66)	10 (8.62)		0.431 (0.169–1.100)	25 (21.55)	2 (1.72)	0.480 (0.366–8.495)	5 (4.31)	22 (18.97)	0.169 (0.059–0.488)**	24 (20.69)	3 (2.59))	1.622 (0.432–6.085)	1 (0.86)	26 (22.41	0.093 (0.012–0.723)**
>40 (89)*	71 (61.21)	18 (15.52)			78 (67.24)	11 (9.48)		51 (43.97)	38 (32.76)		74 (63.79)	15 (12.93)		26 (22.41)	63 (54.31)	
Grade																
1/2 (96)	75 (64.66)	21 (18.10)		1.923 (0.681–5.432)	86 (74.14)	10 (8.62)	1.518 (0.378–6.100)	48 (41.38)	48 (41.38)	1.500 (0.563–3.997)	82 (70.69)	14 (12.07)	1.464 (0.426–5.028)	23 (19.83)	73 (62.93)	1.260 (0.383–4.150)
3 (20)*	13 (11.21)	7 (6.03)			17 (14.66)	3 (2.59)		8 (6.90)	12 (10.34)		16 (13.79)	4 (3.45)		4 (3.45)	16 (13.79)	
Tumor invasion																
T1/T2 (9)	7 (6.03)	2 (1.72)		1.123 (0.220–5.748)	7 (6.03)	2 (1.72)	0.401 (0.074–2.175)	5 (4.31)	4 (3.45)	1.373 (0.349–5.393)	7 (6.03)	2 (1.72)	0.615 (0.117–3.233)	1 (0.86)	8 (6.90)	0.389 (0.046–3.262)
T3/T4 (107)*	81 (69.83)	26 (22.41)			96 (82.76)	11 (9.48)		51 (43.97)	56 (48.28)		91 (78.45)	16 (13.79)		26 (22.41)	81 (69.83)	
Lymph node metastasis																
Absent (6)	4 (3.45)	2 (1.72)		0.619 (0.107–3.575)	5 (4.31)	1 (0.86)	0.612 (0.066–5.689)	4 (3.45)	2 (1.72)	2.231 (0.392–12.686)	5 (4.31)	1 (0.86)	0.914 (0.100–8.318)	2 (1.72)	4 (3.45)	1.700 (0.294–8.832)
Present (110)*	84 (72.41)	26 (22.41)			98 (84.48)	12 (10.34)		52 (44.83)	58 (50.00)		93 (80.17)	17 (14.66)		25 (21.55)	85 (73.28)	
Response to therapy																
Sensitive (50)	46 (39.66)	4 (3.45)		6.571 (2.106–20.509)**	49 (42.24)	1 (0.86)	10.889 (1.365–86.840)**	30 (25.86)	20 (17.24)	2.308 (1.089–4.890)**	48 (41.38)	2 (1.72)	7.680 (1.676–35.199)**	12 (10.34)	38 (32.76)	1.074 (0.451–2.557)
Resistant (66)*	42 (36.21)	24 (20.69)			54 (46.55)	12 (10.34)		26 (22.41)	40 (34.48)		50 (43.10)	16 (13.79)		15 (12.93)	51 (43.97)	
HR expression																
Negative (62)	39 (33.62)	23 (19.83)		0.173 (0.060–0.497)**	50 (43.10)	12 (10.34)	0.079 (0.010–0.627)**	21 (18.10)	41 (35.34)	0.278 (0.129–0.599)**	46 (39.66)	16 (13.79)	0.111 (0.024–0.507)**	8 (6.90)	54 (46.55)	0.273 (0.108–0.691)**
Positive (54)*	49 (42.24)	5 (4.31)			53 (45.69)	1 (0.86)		35 (30.17)	19 (16.38)		52 (44.83)	2 (1.72)		19 (16.38)	35 (30.17)	
HER2 expression																
Negative (67)	62 (53.45)	5 (4.31)		10.969 (3.761–31.981)**	65 (56.03)	2 (1.72)	9.408 (1.979–44.721)**	41 (35.34)	26 (22.41)	3.574 (1.636–7.808)**	65 (56.03)	2 (1.72)	15.758 (3.417–72.663)**	20 (17.24)	47 (40.52)	2.553 (0.981–6.642)**
Positive (49)*	26 (22.41)	23 (19.83)			38 (32.76)	11 (9.48)		15 (12.93)	34 (29.31)		33 (28.45)	16 (13.79)		7 (6.03)	42 (36.21)	
MYC expression																
Negative (80)	77 (66.38)	3 (2.59)		58.333 (15.062–225.913)**	79 (68.10)	1 (0.86)	39.500 (4.883–319.520)**	53 (45.69)	27 (23.27)	21.593 (6.067–76.850)**	78 (67.24)	2 (1.72)	31.200 (6.623–146.981)**	26 (22.41)	54 (46.55)	16.852 (2.187–129.871)**
Positive (36)*	11 (9.48)	25 (21.55)			24 (20.70)	12 (10.34)		3 (2.59)	33 (28.45)		20 (17.24)	16 (13.79)		1 (0.86)	35 (30.17)	
HER2 amplification																
Negative (88)	–	–		–	87 (75.00)	1 (0.86)	65.250 (7.923–537.398)**	54 (46.55)	34 (29.31)	20.647 (4.603–92.614)**	87 (75.00)	1 (0.86)	134.455 (16.267–1111.302)**	26 (22.41)	62 (53.45)	11.323 (1.461–87.758)**
Positive (28)*	–	–			16 (13.79)	12 (10.34)		2 (1.72)	26 (22.41)		11 (9.48)	17 (14.66)		1 (0.86)	27 (23.28)	

* Reference group for logistic regression analysis; ** Differentially expressed between groups, p<0.05. N: number of samples; OR: odds ratio; CI: confidence interval. HR: hormone receptor; CEP17: chromosome 17 signals ; CEP8: chromosome 8 signals; pol8: chromosome 8 polysomy.

HER2 expression and its amplification were associated with HR (p<0.001, OR: 0.162; 95% CI: 0.070–0.373; p = 0.001, OR: 0.173; 95% CI: 0.060–0.497, respectively) and MYC expression (p<0.001, OR: 5.303, 95% CI: 2.255–12.473; p<0.001, OR: 58.333, 95% CI: 15.062–225.913, respectively) (Table 2 and 3).

MYC expression was also associated with HR (p = 0.023, OR: 0.379, 95% CI: 0.164–0.872) (Table 2). *MYC* gain was associated with MYC (p<0.05, for all applied cutoffs), HR (p<0.05, for all cutoffs) and HER2 expression (p<0.05, except for the cut point #4), as well as with *HER2* amplification (p<0.05, for all cutoffs) (Table 3).

*KRAS* codon 12 mutation was observed in 9 (7.76%) tumors. *KRAS* mutation was associated with *HER2* (p = 0.033, OR: 4.565, 95% CI: 1.133–18.39) and *MYC* amplification (p = 0.043, OR: 4.850; 95% CI: 1.049–22.424, for cut point #1) (Table 4).

**Table 4:** Clinicopathological features and protein expression by *KRAS* mutation.

Factor (N)	*KRAS* mutation [N(%)]		OR (95% CI)
	Absent	Present*	
Age			
≤40 (27)	23 (19.83)	4 (3.45)	0.342 (0.085–1.379)
>40 (89)*	84 (72.41)	5 (4.31)	
Grade			
1/2 (96)	91 (78.45)	5 (4.31)	4.550 (1.102–18.788)**
3 (20)*	16 (13.79)	4 (3.45)	
Tumor invasion			
T1/T2 (9)	8 (6.9)	1 (0.86)	0.646 (0.071–5.835)
T3/T4 (107)*	99 (85.34)	8 (6.9)	
Lymph node metastasis			
Absent (6)	5 (4.31)	1 (0.86)	0.392 (0.041–3.775)
Present (110)*	102 (87.93)	8 (6.9)	
Response to therapy			
Sensitive (50)	48 (41.38)	2 (1.72)	2.847 (0.565–14.345)
Resistant (66)*	59 (50.86)	7 (6.03)	
HR expression			
Negative (62)	55 (47.41)	7 (6.03)	0.302 (0.060–1.522)
Positive (54)*	52 (44.83)	2 (1.72)	
HER2 expression			
Negative (107)	63 (54.31)	4 (3.45)	1.790 (0.455–7.043)
Positive (9)*	44 (37.93)	5 (4.31)	
MYC expression			
Negative (80)	76 (65.52)	4 (3.45)	3.065 (0.771–12.176)
Positive (36)*	31 (26.72)	5 (4.31)	
*HER2* amplification			
Negative (88)	84 (72.41)	4 (3.45)	4.565 (1.133–18,390)**
Positive (28)*	23 (19.83)	5 (4.31)	
*MYC* amplification			
*MYC*/CEP8≤2.2 (103)	97 (83.62)	6 (5.17)	4.850 (1.049–22.424)**
*MYC*/CEP8>2.2 (13)*	10 (8.62)	3 (2.59)	

* Reference group for logistic regression analysis; ** Differentially expressed between groups, p<0.05. N: number of samples; OR: odds ratio; CI: confidence interval. HR: hormone receptor; CEP8: chromosome 8 signals.

### The impact of *MYC* and *KRAS* in the molecular phenotype

Taking in account the HR expression and *HER2* amplification to classify tumors by molecular phenotype, 49 (42.2%) of the tumors were deemed luminal A, 5 (4.3%) were luminal B, 23 (19.8%) were HER2 overexpressed, and 39 (33.6%) were triple-negative.

MYC expression was associated with luminal B and HER2 overexpression phenotypes (p = 0.008, OR: 24, 95% CI: 2.329–247.368; p<0.001, OR: 63, 95% CI: 12.021–330.170; respectively) compared to luminal A. The presence of *MYC* duplication or polysomy 8 (cut point #2) was also associated with the HER2 overexpression subtype (p<0.001, OR: 49.867, 95% CI: 6.143–404.814).

*KRAS* mutation was detected in 1/49 (2%) luminal A, 1/5 (20%) luminal B, 4/23 (17.4%) HER2 overexpression and 3/39 (7.7%) triple-negative tumors. *KRAS* mutation was associated with HER2 overexpression phenotype in relation to luminal A (p = 0.044, OR: 10.105, 95% CI: 1.06–96.336).

### The impact of protein and gene status on clinicopathological features

MYC expression and *MYC* gain were more frequently observed in early-onset compared to late-onset tumors (p = 0.002, OR: 0.247, 95% CI: 0.100–0.610; p<0.05, for cutoffs #2 and #4; respectively) (Table 2 and 3).

The expression of HR expression presented a protective effect for grade 3 tumors (p = 0.014, OR: 0.23, 95% CI: 0.072–0.738) (Table 2). On the other hand, HER2 expression and *KRAS* mutation was a risk factor for grade 3 tumors (p = 0.008, OR: 4.067, 95% CI: 1.433–11.537; p = 0.036, OR: 4.55; 95% CI: 1.102–18.788, respectively) (Table 2 and 4). Since HR and HER2 expression were associated with grade 3 tumors, logistic regression was also performed using the molecular phenotype as dependent variables. Women with HER2 overexpression and triple-negative tumors (p = 0.027, OR: 5.412, 95% CI: 1.216–24.094; p = 0.017, OR: 5.287, 95% CI: 1.342–20.836, respectively) had elevated risk of being diagnosed with grade 3 tumors relative to those with luminal A tumors.

The logistic regression model performed to verify if molecular were together associated with the risk of grade 3 tumors showed that the final model only included *KRAS* mutation.

### The impact of protein and gene status on chemotherapy response

The overall response rate of primary tumor to preoperative chemotherapy was 43%. Among responsive patients, only 4 (8%) patients died at the end of this study (minimum follow-up time of over 12 months). These patients presented metastatic tumors about 2 years after the treatment for primary cancer.

The expression of HR presented a protective effect for treatment-resistance (p<0.001, OR: 0.042, 95% CI: 0.016–0.113) (Table 1). On the other hand, HER2 expression and its gene amplification were a risk factor for chemotherapy resistance (p<0.001, OR: 8.610, 95% CI: 3.483–21.283; p = 0.001, OR: 6.571, 95% CI: 2.106–20.509, respectively) (Table 2 and 3). Additionally, *HER2* overexpression (p<0.001, OR: 46.67, 95% CI: 9.229–235.97) and triple-negative (p<0.001, OR: 24.44, 95% CI: 7.887–75.759) subtypes presented an increased risk of being resistant to chemotherapy relative to luminal A.

MYC expression and its gene amplification were a risk factor for chemotherapy resistance (p = 0.01, OR: 3.154, 95% CI: 1.318–7.547; p<0.05, except when the cut point #4 was applied; respectively) (Table 2 and 3).

We conducted a forward stepwise logistic regression model to identify predictors of chemotherapy resistance, entering age, stage, grade, molecular phenotype, *KRAS* mutation, MYC expression, and the FISH results for detection of its amplification (including the different cutoffs described above for *MYC* status) as dependent variables. The OR was calculated considering the treatment-resistant group in relation to chemotherapy sensitivity group. The final model included *MYC* amplification defined as *MYC*/nucleus ratio of ≥2.5 (cut point #4; p = 0.016, OR: 0.109, 95% CI: 0.018–0.664) as a protective factor. On the other hand, age (p = 0.02, OR: 1.063, 95% CI: 1.01–1.12) was a risk factor. Additionally, luminal B, HER2 overexpression, and triple-negative tumors (p = 0.006, OR: 42.063, 95% CI: 2.956–598.51; p<0.001, OR: 172.754, 95% CI: 15.754–1894.386; p<0.001, OR: 49.008, 95% CI: 8.789–273.268, respectively) presented increased odds of being resistant to chemotherapy relative to luminal A tumors. Moreover, grade 2 tumors presented an increased risk of being resistant to treatment relative to grade 1 (p = 0.042, OR: 10.544, 95% CI: 1.087–102.252). However, grade 3 tumors did not present an increased risk relative to grade 1 in this model.

## Discussion

In this study, we evaluated ER and PR expression, *HER2* and *MYC* genes and their protein status, and *KRAS* mutations in the same set of BC. First, we observed that 24% of tumors presented *HER2* amplification, corroborating a previous study (18–20%) [26]. *HER2* amplification is the primary mechanism of HER2 overexpression [27]. Although an excellent concordance between IHC and FISH results was detected, 5 cases were scored as 1+ by IHC and presented HER2 amplification. Since standardization of IHC tests may be affected by preanalytical and analytical factors, some groups have suggested the utilization of FISH results for HER2 protein overexpression determination in BC [25]. Therefore, we used only the FISH result for HER2 in the molecular phenotype classification and, then, in the multivariate analyses.

Several definitions for *MYC* amplification have been used in BC studies. However, these different definitions lead to inconsistent results concerning the role of *MYC* in breast carcinogenesis. Here, we applied different cutoffs to define *MYC* amplification as described above, including the acceptance of low *MYC* gain with or without polysomy 8. The frequency of *MYC* amplification ranged from 11.2% (cut point #1) to 76.7% (cut point #4) in our sample. Furthermore, MYC overexpression was detected in 31% of BC. Although we found an association between *MYC* amplification and its expression, as already described in previous studies, our data confirm that mechanisms other than gene amplification are involved in MYC overexpression in BC [9]. However, the assessments of MYC expression by IHC provide variable results depending on the antibody, testing protocol, and scoring system used [10], highlighting that FISH may be an interesting tool in clinical practice due to its reproducibility.

We observed that *MYC* amplification and expression were more frequent in BC without ER or PR expression, corroborating previous studies [23]. However, some investigations did not find such inverse correlation or even show the opposite correlation (see review [28]). Although literature findings are inconsistent regarding associations between *MYC* amplification and clinicopathological parameters (in part, probably due to the lack of a unique cutoff for *MYC* amplification definition), a meta-analysis demonstrated that the correlation of *MYC* amplification with PR negativity was the only statistically significant association [29].

Here, we detected an association between *MYC* and *HER*2, as previously reported [30], [31]. As expected, we also observed an association between MYC expression or its amplification with luminal B or HER2 overexpression in relation to luminal A, confirming the results for *HER2* and HR described above. These findings suggest that MYC may be involved in subtype-specific pathways.

MYC expression and gain were more frequently observed in early-onset compared to late-onset BC. To our knowledge, this is the first study to report this association in human primary BC. The exact mechanisms by which MYC may be involved in early-onset BC needs to be elucidated. However, *MYC* amplification seems to be associated to *BRCA1* inactivation in a group of hereditary and sporadic BC [32]. *BRCA1* inactivation is usually predisposed to early-onset tumors, with a distinct phenotype characterized by high tumor grade, aneuploidy, high proliferation rate, and ER-negativity [32]. The investigation of MYC targets is still necessary to better understand the heterogeneity of BC.

*MYC* amplification, as well as *HER2* amplification, was associated with *KRAS* codon 12 mutation. In BC cells, KRAS may be activated by HER2 [1], enhancing the accumulation of MYC activity [11], which may lead to chromosomal instability [33] and contribute to *MYC* amplification. *KRAS* mutation was detected in 7.76% of the tumors, corroborating a previous study which reported that 5% of BC presented some *KRAS* mutation [34]. More than one case of HER2 overexpression and triple-negative subtypes presented *KRAS* mutation. However, previous studies did not find any *KRAS* mutation in triple-negative tumors, probably due to its low frequency [35], [36]. Thus, an increased number of tumors are essential to provide evidence of the role of *KRAS* in human breast carcinogenesis.

In our population, HER2 overexpression and triple-negative were more frequently grade 3 tumors, which is in agreement with the more aggressive phenotype of these tumors [3], [4]. Additionally, we observed that the *KRAS* mutation was the main predictive factor for grade 3 tumors. Due to its small frequency, further investigations are still necessary to evaluate whether a *KRAS* codon 12 mutation may predict a worse prognosis in BC patients.

Concerning the response to chemotherapy, we observed that HR predicts chemosensitivity, as previously reported [1]. On the other hand, HER2 amplification or expression predicts resistance to anthracyclines in our sample, highlighting that this group of patients may be suitable for treatment with the monoclonal antibody trastuzumab [37].

Furthermore, MYC expression and amplification (except when accepting low *MYC* gain) was a risk factor for chemoresistance by univariate logistic regression. However, in the multivariate analysis to identify predictors of resistance to doxorubicin plus cyclophosphamide drugs, we observed that *MYC* amplification (including low ratio of *MYC* gain) was a predictor of chemosensibility when adjusted by grade, age, and the molecular phenotyping. This finding is probably due to the significant association of MYC with both HR (good prognosis) and HER2 (worse prognosis).

MYC may have a dual function in cancer cells, i.e. it can promote cell proliferation or induces apoptosis [8] depending on molecular background and tumor microenvironment. Since rapidly proliferating cells are generally more sensitive to chemotherapy, it has been suggested that MYC may sensitive BC cells to apoptosis [38]. Previous *in vitro* studies demonstrated greater sensitivity of BC cells with *MYC* amplification to paclitaxel and to doxorubicin compared to those without this amplification [39], [40], [41]. To our knowledge, few studies evaluated the possible role of MYC as a predictor for chemotherapy response in humans. Yasojima et al. reported that *MYC* was associated with the response to neoadjuvant chemotherapy comprising paclitaxel followed by 5-FU/epirubicin/cyclophosphamide by univariate analysis. However, the multivariate analysis failed to show such association [38]. Without performing a multivariate analysis, Todorovic-Rakovic et al. suggested that patients with *MYC* amplification treated with chemotherapy (cyclophosphamide, methotrexate and 5-fluorouracil or 5-fluorouracil, adriablastin and cyclophosphamide) had clinical benefits in contrast to patients without amplification [7]. Furthermore, Perez et al. reported that tumors with *MYC* gain or polysomy 8 appeared to derive more benefits from trastuzumab than tumors without these alterations. The author also reported that patients with *MYC*/*HER2* coamplification benefited significantly more from trastuzumab than patients with only *HER2* amplification [23]. Our results and those from the literature suggest that *MYC* amplification may be used as a predictor factor for chemosensibility and treatment determination. However, it is important to evaluate several markers concomitantly to try to determine a statistical model to identify patients who would best respond to a treatment.

As a result of the increased number of chemotherapy regimens that have been applied in the preoperative setting, it is becoming increasingly important to identify patients who carry a particularly high risk for being unresponsive for a specific treatment. To our knowledge, this is the first study to evaluate the possible prognostic and predictive significance of *MYC* and *KRAS* alterations concomitantly with HR and HER2 status, in BC patients treated with neoadjuvant doxorubicin plus cyclophosphamide drugs. We observed an association among the molecular markers investigated. BC with *KRAS* codon 12 mutations seem to present a worse prognosis. Additionally, *MYC* amplification may help in the identification of tumors that are sensitive to doxorubicin plus cyclophosphamide. If confirmed in a large set of samples, these markers may be useful for clinical stratification and prognosis.
